# The Implications of Type 1 Diabetes Mellitus Associated with Coeliac Disease

**DOI:** 10.3390/jcm14145129

**Published:** 2025-07-18

**Authors:** Geoffrey Holmes, Peter Gillett

**Affiliations:** 1 Department of Gastroenterology, Royal Derby Hospital, Derby DE22 3NE, UK; 2 Department of Paediatric Gastroenterology, Royal Hospital for Children and Young People, Edinburgh EH16 4TJ, UK; peter.gillett@nhs.scot

**Keywords:** coeliac disease, type 1 diabetes mellitus, association, screening

## Abstract

T1D and CD commonly occur together. This association has received increasing attention from researchers and is considered in detail in this review. Since CD is over-represented in T1D, it may cause ill health with attendant complications, but because there is an effective dietary treatment, screening has been recommended in children and adults. However, there are many unknowns regarding this association, and understanding the why, when, and how with regard to screening and managing those with dual diagnoses requires thorough consideration when introducing the concept of screening to patients. It is important that patients and, where appropriate, carers are put at the heart of the decision-making process with careful discussion of the issues involved before undertaking screening that might uncover a second life-changing diagnosis, for which, without preparatory preparation and support, individuals may be ill-prepared, causing mental health issues. For some patients, an initial policy of monitoring rather than moving to immediate small bowel biopsy and exposure to a gluten-free diet (GFD) will be appropriate. The correct management of patients will ultimately improve their quality of life medically and socially.

## 1. Introduction

If patients with CD are to be treated optimally, potential complications or associated disorders must also be diagnosed and treated without significant delay. The number of comorbidities has mushroomed in recent decades, and many reports continue to be published. Some comorbidities will occur coincidentally, but others will have a statistically valid link, while others can be regarded as complications of CD. Publication numbers are very extensive, and earlier reports have been summarised in detail [1,2,3]. T1D, autoimmune thyroid disease (AITD), inflammatory bowel disease, rheumatoid arthritis, and psoriasis make up about 95% of coexistent immune-mediated conditions associated with CD [4]. There is increasing attention on screening for the “big three” of CD, T1D, and AITD [5,6,7]. Diabetes was the earliest disorder to be reported in a child with CD in 1925 [8] and in an adult in 1956 [9], and this association continues to receive the most attention from researchers and forms the basis of this review.

## 2. Prevalence

The prevalence of both CD and T1D has risen markedly in recent decades, and environmental considerations must be responsible because genetic factors do not readily explain this occurrence [10,11]. It has been known for many years that the diagnosis of T1D is over-represented in CD [12]. A prevalence of 4.5% was found when 26 reports from 1984 to 2002 were analysed [13]. This has been amply confirmed [14,15,16,17]. A pooled analysis of 55 studies comprising 71,853 subjects showed that the prevalence of biopsy-proven CD in T1D was 4–5% depending on the method of calculation. A subgroup analysis revealed a higher prevalence in Asia than in America or Europe [18]. A further meta-analysis involving data from 65,102 individuals with T1D from 106 publications from 40 countries found a pooled prevalence of confirmed CD of 6% [19]. Differences in confirmed CD across continents were not statistically significant, but across countries in Asia, for example, Saudia Arabia (11%) and India (10%) had amongst the highest prevalence rates, but importantly, there were also high-quality systematic, biopsy-proven CD diagnostic studies from Denmark (8.5%) and Sweden (9.1%) [11]. This may be due to genetic factors and diets rich in gluten-containing foods, or potentially due to more systematic screening programmes such as those that exist in Scandinavia, other European countries, and in the UK. Another example is a study from Jordan comprising 538 children with T1D that found that 16.6% were serology-positive but 9.1% had biopsy-proven CD [20]. The risk of developing CD appears to be threefold higher in children diagnosed with T1D before the age of four years compared with those diagnosed before the age of nine [21]. In contrast to T1D, the prevalence of type 2 diabetes (T2D) is similar to that of the general population [12,15,22] or even lower [23].

## 3. Pathogenesis

Genetic and environmental factors play a part, although not yet fully worked out, in the coexistence of T1D and CD.

### 3.1. Genetic Background

The HLA genotype DQ2.5/DQ8 confers a high risk of developing T1D and CD (double immunity). The non-HLA genes CTLA4 and IL2RA also appear to be implicated in double immunity [24]. First-degree relatives of those with T1D are prone to developing CD and AITD, indicating a role for the presence of susceptibility genes [25,26].

### 3.2. Role of Gluten

A study from Italy concluded that the prevalence of autoimmune disorders associated with CD, including T1D, is related to the length of exposure to gluten [27], but this was not confirmed [28] and remains contentious [29]. A possible role of gluten in the development of both types of diabetes has been summarised [30,31]. Mechanisms may include an effect on the intestinal microbiota, intestinal permeability, and inflammation in the pancreas. A Danish study showed that a high gluten intake in mothers during pregnancy increased the risk of their offspring developing T1D. Those having the highest gluten intake compared with those with the lowest had twice the risk of their children having diabetes [32]. NOD mice fed a lifelong GFD almost completely prevented diabetes in their offspring, but no such association has been found in human studies [32]. One investigation found that gluten intake is inversely associated with T2D risk [33].

### 3.3. Infections

A prospective study provided evidence that rotavirus may increase the risk of CD in children [34], but this has not been proven [35], and it does not seem to play a part in the development of T1D [36]. There is an association between enterovirus and T1D [37]. Viruses do not seem to be implicated in the coexistence of CD and T1D. Studies of the microbiome and metabolome are key in the evolving understanding of aetiology and pathogenesis [38,39].

### 3.4. Clinical Aspects

An early review indicated that most children who have T1D do not complain of gastrointestinal symptoms at the time of screening for CD, but gastrointestinal disturbances, failure to thrive, short stature, and delayed puberty may occur, and hypoglycaemia can be a symptom [13]. Adults may also be asymptomatic or have symptoms of undiagnosed CD, such as diarrhoea, bloating, and lethargy. Recurrent hypoglycaemia can be a presenting symptom [40]. CD impaired linear growth and weight in children with T1D [41]. In various reports, the number of patients with symptoms and signs of CD differs widely, probably reflecting how carefully these were sought. It is likely that some patients were labelled as asymptomatic when they were not. In addition, ill health may only be recognised retrospectively following the benefits conferred by adopting a GFD. Nevertheless, these studies indicate the wide range of disturbances, both clinical and laboratory, that those patients with coexistent T1D and CD may experience and how easy it can be to overlook these.

A survey employing large databases from Sweden found that having a diagnosis of CD did not appear to influence the risk of hypoglycaemia, ketoacidosis, or coma in those with T1D [42]. The emergence of pump therapy and continuous glucose monitoring (CGM) may allow a more in-depth investigation on pre-, peri-, and post-CD diagnosis effects on glycaemic control and potentially renal and cognitive function.

Having concomitant diabetes of both types increased the risk of hypertension and coronary heart disease. Those with T1D were less effective at adhering to a GFD [15]. Concomitant CD and T1D do appear to significantly increase the risk of developing AITD [43]. Of 90 such patients, 54 developed AITD. Patients were at risk of both hypo- and hyperthyroidism. There is a case for screening these individuals for thyroid disease because of a reduction in quality of life that they might experience. A high prevalence of microvascular complications, comprising nephropathy and retinopathy, in adults with T1D and CD has been discovered, but their mechanisms are not known [44]. The risk of osteoporosis is increased in T1D alone [45] as it is in CD [46], but longitudinal data are scarce, and there is a need to identify risk in the combined conditions. Studies employing large databases from Sweden found an increased risk of death in those with T1D and CD compared with patients who had T1D alone [47] but no increased fracture risk [48] or risk of renal disease [49].

### 3.5. Adherence to GFD

Adherence to a GFD is variable and brings multifactorial issues, along with variable benefits and challenges with a dual diagnosis. An investigation of young people (8–18 years) with T1D and CD who did not adhere to a strict GFD had a lower quality of life and worse glycaemic control compared to those with T1D only, indicating that strategies to improve dietary adherence in this group are needed [50]. In a small prospective, randomised controlled trial, there was a trend towards a decrease in hypoglycaemic episodes and better glycaemic control in those with subclinical CD and T1D on a GFD [51]. A study of Swedish children with T1D and CD found that only 68% had good compliance to a GFD compared with 85–96% in large samples of children with CD only [52]. Others have provided similar figures [53]. Poor compliance was more evident in older children and those with poor glycaemic control, indicating that this group requires more intensive dietary support. A systematic review of 20 studies revealed that adherence to a GFD in young people with T1D and CD had beneficial effects on clinical and psychological outcomes, growth, glycaemic control, lipid profiles, and quality of life [54], but another review suggested little overall benefit in glycaemic control, microvascular issues, and also challenges with adherence to a GFD [11]. There is a clear need for larger prospective studies to determine the short-, medium-, and long-term benefits and challenges.

### 3.6. Screening At-Risk Patients

Since CD is over-represented in T1D, there are reliable serological tests for CD. It can cause significant symptomatic ill health with typical gastrointestinal and non-gastrointestinal symptoms, and there is an effective dietary treatment, so screening is recommended in children [55] and adults [56]. Screening studies in those at risk for T1D (pre-diabetes) are based on delaying the presentation of T1D, education regarding the condition, understanding how to eventually deal with it by recognizing the symptoms early, and reducing the harmful effects of abnormal glycaemic control, particularly ketoacidosis, on cognition and the developing brain [57,58]. However, this enthusiasm for screening has been tempered by others as detailed below.

An investigation from Ireland of 253 children and adolescents with T1D screened for CD by anti-tissue transglutaminase (tTG) found that 37 (14.6%) were positive, of whom 26 (70.3%) tested positive in the first 2 years after the diagnosis of T1D [59]. In addition, all 37 had endomysial antibodies (EMA) when tested, and 35 were positive (94.6%). It was suggested that the first screening should be 6–12 months after the diagnosis of diabetes. The timing for subsequent screenings was unclear. Screening was not recommended after 6 years because all cases of CD were diagnosed during the first 6 years after the diagnosis of T1D [60], but others have extended this time to 10 years [61]. In practice, clinicians will likely begin screening around the time of the diagnosis of T1D and periodically for perhaps 10 years or at any time if there are clinical indications such as a decline in growth or gastrointestinal symptoms indicative of CD. The case for screening is established in the minds of most practitioners. A national paediatric screening programme for T1D and CD is envisioned for Italy, and while the details are yet to be worked out, this is an exciting development [6,62].

### 3.7. Making the Diagnosis of CD in T1D

In the 2012 European Society for Pediatric Gastroenterology, Hepatology, and Nutrition (ESPGHAN) guidelines for the diagnosis of CD, an algorithm indicates that children with a high risk for CD who are asymptomatic with an anti-tTG antibody level greater than three times the upper limit of normal should have a small bowel biopsy performed [63]. Guidelines emanating from the British Society of Paediatric Gastroenterology, Hepatology, and Nutrition (BSPGHAN) made similar recommendations when published in 2013 [64]. A revision of the ESPGHAN guidelines in 2020 states that more data from high-quality studies in children with diabetes with regard to a no-biopsy approach is needed [65]. Consideration of testing for HLA-DQ in the screening process is not currently recommended because the proportion of DQ negatives, and therefore the chance of not having CD, is very small, and it is not a cost-effective strategy [65,66,67,68]. A recent study on high anti-tTG IgA and positive EMA in patients with T1D confirms that high levels over 10 times the upper limit of normal are consistent with histological evidence of CD, whereas lower levels are less consistent [69]. Whether patients with T1D have histological changes in the same degree needs to be properly researched. There is no doubt that all selective IgA-deficient (sIgAD) patients, including those with T1D with positive anti-tTG IgG antibodies, need to be biopsied to fully understand the histological–serological correlation in this subgroup [70].

Positive anti-tTG antibodies were found in 103 (15.4%) of 668 children and adolescents with T1D [71]. However, of these, 24 (23.3%) normalised spontaneously while on a normal diet within a median duration of nine months. The median follow-up was 25.5 months (6–105). Those who normalised were all asymptomatic, and the median antibody levels were initially low in most. It was concluded that in those who are asymptomatic and with low anti-tTG levels, monitoring, rather than immediate biopsy, and a GFD are appropriate. Others have also noted a high rate of the spontaneous normalisation of coeliac serology in some children with coexistent T1D [72]. Those with high or moderately high levels of serology were less likely to normalise spontaneously than those with low levels. Again, this indicates that asymptomatic patients with only mild or low levels could have regular serological follow-up tests rather than be rushed into having an endoscopy and unnecessary exposure to a GFD. A study employing a large database of children with CD and T1D found that those who are asymptomatic can have biopsies and the initiation of a GFD delayed for up to three years without compromising health [73]. In some patients, EMA may also normalise spontaneously [74].

Deferring testing may be contemplated on an individual basis, considering factors such as family circumstances and the immediate ability of a young person to cope with having two disorders, as well as their level of symptoms and the potential for a spontaneous resolution of serology. Just because a course of action can be taken does not mean that it should be taken.

Having CD and T1D separately is burdensome, and more so for both disorders together. Diabetes distress refers to the emotional effects of living with diabetes and may include feelings of guilt and anxiety, as well as concerns about self-managing the disorder. This is common, with an estimated prevalence of more than 20%, for both T1 and T2D. Interventions in this area are available but have not yet been rolled out systematically across the UK [75]. Patients with diabetes have impaired quality of life that extends to thoughts of suicide, although suicidal acts are uncommon [76,77,78,79]. Those with CD can also suffer from quality-of-life issues [80,81,82,83] and burdens associated with a GFD [84]. Adding a GFD to those with T1D can impose further restrictions on lifestyle, and in addition, failure to adhere to the diet among those with T1D and CD is common, with only about 60% compliant compared with about 80% for CD alone [53].

### 3.8. Dilemmas and Solutions

Thus, there are key questions that require consideration when introducing the concept of screening to patients and their carers. Parents, children, and young people now have easy access to information, which may not always be reliable, through the internet and other sources, and they research issues carefully for themselves. They often ask searching questions, such as the following: What are the implications for me if I am screened? What does the early detection of a condition achieve? All the complications and associations (some relatively rare) that may arise in undiagnosed CD (and detailed on patient organisation websites) such as osteoporosis, problems in reproductive health, the development of other AI diseases and dermatitis herpetiformis, malignancy, particularly lymphoma, and neurological issues are all asked about and are in urgent need of specific research especially in this most important group of patients. The reported malignancy risk is limited to historical case reports, for example, where four patients with T1D (who had not been previously screened) developed lymphoma [85]. More modern registry data (though not specific to those with T1D and CD) show no or limited increased risk [86,87]. Data specifically relating to patients with T1D/CD (and which does not differentiate symptomatic versus asymptomatic patients) for relevant long-term risks is scarce, and this also relates to growth, BMI, glucose control, and overall HbA1C post-T1D diagnosis. Clinicians must have clear answers, wherever possible, to these questions and be clear about what level of risk is involved in pursuing or not pursuing diagnoses either immediately or later. The universal principle in medicine should apply, such as to first do no harm, in this steadily evolving process, and we need clearer evidence as to what difference GFD makes in truly asymptomatic patients [88].

**Figure 1:**
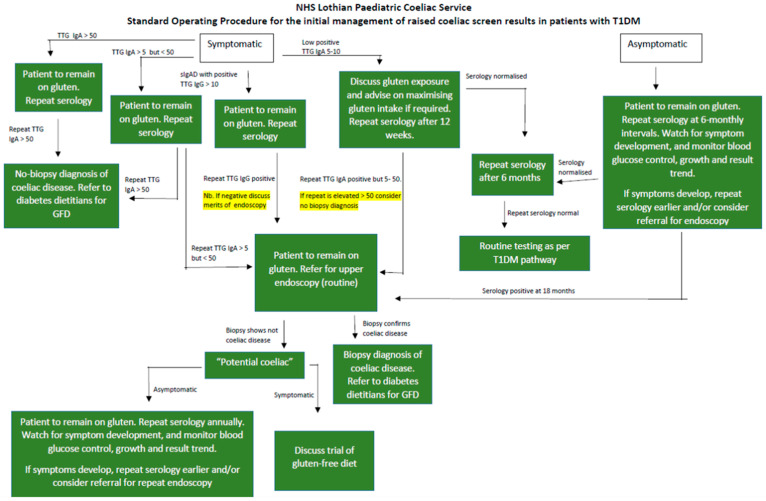
NHS Lothian Paediatric Coeliac Service Standard Operating Procedure for the initial management of raised coeliac screen results in patients with T1DM.

This is how the NHS Lothian paediatric T1D and coeliac services approached these issues in the 1990s after much discussion regarding both conditions and the emerging increased mental health problems encountered around the whole process of diagnosis and screening. The process was changed by pragmatically watching and waiting, repeating blood tests, and monitoring clinical reasons for endoscopic assessment. Guidelines and local pathways were created for all to follow (Figure 1). In view of all of this, there is a need to temper the enthusiasm for screening too soon and revisit the strong recommendations from NICE from 2015 regarding testing for CD at diagnosis of T1D. There is a risk of such screenings taking place without any or inadequate discussion with the patient or family, as well as subsequent referrals for endoscopy or GFD without a full understanding from health care professionals about management options or the natural history of the disorders. Since 2020, ESPGHAN’s no-biopsy guidance may also have been over-interpreted to include asymptomatic patients with T1D, which was not the group’s intention [89,90].

## 4. Future Considerations

Interestingly, priorities for patients with T1D in a recent James Lind Alliance top 10 did not have CD as an important issue [91]. This goes against the position of Coeliac UK, which in 2018, in their priority setting partnership, rated associated T1D and AITD as a high priority and placed this topic at number 5 out of 10 others that were considered [92]. There are still many unknowns regarding the CD and T1D association, as highlighted in this review. Perhaps it is time to put children and families more at the heart of the decision-making process and move to an opt-in approach for those who are truly asymptomatic or at least consider testing in a more thoughtful manner. Careful discussion of the issues is necessary with patients and families before undertaking screening. How consent is obtained for screening is crucial for what might be a life-changing diagnosis with very far-reaching implications. A watch-and-wait approach has been shown to be entirely appropriate for some patients.

To answer important research questions, collaborative working should be a priority and is more productive than groups working in isolation. This would allow large numbers of patients and control subjects to be recruited for necessary studies to illuminate this association. Many reports are based on small numbers of patients followed for only short time periods, making valid statistical analysis impossible. Key issues for clinicians and researchers are to develop an understanding of the differences in risk for those who are symptomatic against those who are not, as well as the support, both clinical and psychological, that patients need to cope optimally with their problems. There is an urgent requirement, particularly for long-term follow-ups of large numbers of patients, to better understand the natural history of the association between CD and T1D.

## 5. Conclusions

While much progress has been made in understanding the CD and T1D association in its recognition, in addition to national advice given by NICE NG20 [90] and Quality Standard 134 [89] recommendations from 10 years ago regarding screening and as per the 2022 guidance from ISPAD [93], there needs much more thought from both gastroenterologists and endocrinologists as to why, when, and how to screen and how to manage those affected by the dual diagnosis. It is clear that we need better evidence on the risks of not diagnosing CD in T1D and in demonstrating the benefits of screening and managing children and adults who consider themselves asymptomatic, with respect to complications, and whether those risks differ depending on the presentation of CD. There is little doubt that the correct management of these patients will result in improvements in their well-being and quality of life medically and socially.
